# Overexpression of MicroRNAs from the miR-17-92 Paralog Clusters in AIDS-Related Non-Hodgkin's Lymphomas

**DOI:** 10.1371/journal.pone.0020781

**Published:** 2011-06-16

**Authors:** Dharma R. Thapa, Xinmin Li, Beth D. Jamieson, Otoniel Martínez-Maza

**Affiliations:** 1 Department of Microbiology, Immunology and Molecular Genetics, David Geffen School of Medicine at UCLA, University of California Los Angeles, Los Angeles, California, United States of America; 2 Department of Pathology and Laboratory Medicine, David Geffen School of Medicine at UCLA, University of California Los Angeles, Los Angeles, California, United States of America; 3 Department of Medicine, David Geffen School of Medicine at UCLA, University of California Los Angeles, Los Angeles, California, United States of America; 4 Department of Obstetrics & Gynecology, David Geffen School of Medicine at UCLA, University of California Los Angeles, Los Angeles, California, United States of America; 5 Department of Epidemiology, UCLA School of Public Health, and UCLA AIDS Institute and Jonsson Comprehensive Cancer Center, University of California Los Angeles Los Angeles, California, United States of America; Universität Würzburg, Germany

## Abstract

**Background:**

Individuals infected by HIV are at an increased risk for developing non-Hodgkin's lymphomas (AIDS-NHL). In the highly active antiretroviral therapy (HAART) era, there has been a significant decline in the incidence of AIDS-associated primary central nervous system lymphoma (PCNSL). However, only a modest decrease in incidence has been reported for other AIDS-NHL subtypes. Thus, AIDS-NHLs remain a significant cause of morbidity and mortality in HIV infected individuals. Recently, much attention has been directed toward the role of miRNAs in cancer, including NHL. Several miRNAs, including those encoded by the miR-17-92 polycistron, have been shown to play significant roles in B cell tumorigenesis. However, the role of miRNAs in NHL in the setting of HIV infection has not been defined.

**Methodology/Principal Findings:**

We used quantitative realtime PCR to assess the expression of miRNAs from three different paralog clusters, miR-17-92, miR-106a-363, and miR-106b-25 in 24 cases of AIDS-NHLs representing four tumor types, Burkitt's lymphoma (BL, n = 6), diffuse large B-cell lymphoma (DLBCL, n = 8), primary central nervous system lymphoma (PCNSL, n = 5), and primary effusion lymphoma (PEL, n = 5). We also used microarray analysis to identify a differentiation specific miRNA signature of naïve, germinal center, and memory B cell subsets from tonsils (n = 4). miRNAs from the miR-17-92 paralog clusters were upregulated by B cells, specifically during the GC differentiation stage. We also found overexpression of these miRNA clusters in all four AIDS-NHL subtypes. Finally, we also show that select miRNAs from these clusters (miR-17, miR-106a, and miR-106b) inhibited p21 in AIDS-BL and DLBCL cases, thus providing a mechanistic role for these miRNAs in AIDS-NHL pathogenesis.

**Conclusion:**

Dysregulation of miR-17-92 paralog clusters is a common feature of AIDS-associated NHLs.

## Introduction

The risk for developing non-Hodgkin lymphoma (NHL) is greatly increased in those persons who are living with HIV infection. In fact, NHL in the setting of HIV infection, is an AIDS defining condition. AIDS-related NHL comprise a heterogenous group of B cell lymphomas that includes Burkitt's lymphoma (BL), diffuse large B-cell lymphoma (DLBCL), primary central nervous system lymphoma (PCNSL), primary effusion lymphoma (PEL) and plasmablastic lymphomas (PBL) [1]. In the post-HAART era there has been a dramatic reduction in the incidence of PCNSL, however, only a modest decrease has been reported for other NHL subtypes [2], [3]. Various factors are believed to contribute to the pathogenesis of AIDS-NHL, including immune deficiency, chronic B cell stimulation, cytokine dysregulation, genetic lesions involving oncogenes or tumor suppressor genes, and the presence of Epstein-Barr virus (EBV) and/or Kaposi's sarcoma-associated herpesvirus/human herpesvirus-8 (KSHV/HHV-8) [4].

microRNAs (miRNAs) are a class of endogenous small non-coding RNAs (∼21–23 nt) that bind to the 3′ untranslated region (UTR) of mRNAs and mediate cleavage or cause translational inhibition [5]. miRNAs are conserved and expressed across diverse species including animals, plants, and viruses [6]. So far, 940 human miRNAs (Sanger miRBase v15) have been identified in humans and they regulate critical cellular processes including proliferation, differentiation, metabolism, cell death/apoptosis and tumorigenesis (reviewed in [7]). miRNAs can also act as tumor suppressors or oncogenes and have been shown to be deregulated in almost every tumor type studied. Furthermore, miRNA profiling has revealed tumor specific signatures with diagnostic, prognostic, and therapeutic implications [7].

It is becoming increasingly clear that miRNAs play a role in B cell tumorigenesis. miR-16-1/miR-15a cluster located at 13q14, is frequently deleted or downregulated in chronic lymphocytic leukemia (CLL), leading to upregulation of its target protein Bcl2 [8]. Loss of miR-16-1 binding sites due to translocation of *CCND1* (cyclin D1) and truncation of its 3′UTR contributes to its overexpression in mantle cell lymphomas (MCL) [9]. Additionally, miR-155, encoded by exon 3 of the non-coding *BIC* (B cell integration cluster) transcript [10], is upregulated in Hodgkin's lymphoma [11], [12], primary mediastinal B cell lymphomas [11], and DLBCL [11], [13], but not in BL [14]. Accordingly, transgenic overexpression of miR-155 in mouse model leads to development of pre-B cell leukemia and ultimately high grade lymphomas [15]. Even a B cell tropic oncogenic herpesvirus, EBV, has been shown to upregulate miR-155 [16], whereas HHV-8 encodes miR-K12-11, which is an ortholog of human miR-155 [17], [18].

Several studies have suggested an oncogenic role for the miR-17-92 cluster (which encodes seven miRNAs: miR-17-5p, miR-17-3p, miR-18a, miR-19a, miR-20a, miR-19b-1, and miR-92-1) in B cell lymphomagenesis. Chromosomal amplification at 13q31-q32 leads to overexpression of the miR-17-92 cluster encoded by the *Chromosome 13 open reading frame 25* (*C13orf25*) gene in several B cell lymphomas, including DLBCL [19]–[21], follicular lymphoma [21], [22], mantle cell lymphoma [23], and primary cutaneous B-cell lymphomas [24]. Consistent with its proposed oncogenic role, enforced expression of the miR-17-19b polycistron greatly accelerated lymphoma onset in mouse model of lymphoma driven by *c-myc* expression [21]. miR-17-92 is also overexpressed in *MLL* (mixed lineage leukemia)-rearranged acute myeloid leukemia (AML) and acute lymphoblastic leukemia (ALL) [25], and in CD34+ chronic myeloid leukemia (CML) [26]. Overexpression of the miR-17-92 cluster has been shown to enhance proliferation in CML lines [26], BL and DLBCL lines [27], and to play a role in a mouse model of MLL-leukemia [28], by targeting the cell cycle inhibitor p21. In the *c-myc* induced B-cell lymphoma mouse model, miR-19a and miR-19b were shown to be largely sufficient for the oncogenic property of miR-17-92 cluster, operating by targeting the tumor suppressor PTEN [29]. Finally, miR-17-92 cluster was also shown to target the proapoptotic protein Bim, in a mouse model, with overexpression [30] or deletion of the cluster [31].

Even though HIV does not directly infect B cells, HIV infection is associated with a marked increase in risk for NHL [32]. In contrast to NHLs that develop in immunocompetent hosts, AIDS-NHLs typically exhibit a more aggressive clinical phenotype with a predilection for extranodal sites [1]. The role of miRNAs in B cell lymphomas in the setting of HIV/AIDS and immunodeficiency is not well understood. Here we asked if miRNA overexpression from the miR-17-92 cluster, as well as its two other paralogs (miR-106a-363, and miR-106b-25), is also a common feature of B cell lymphomas in the setting of HIV infection and AIDS. Using quantitative real-time PCR (q-PCR), we assessed the expression of miRNAs from these three paralog clusters in 24 cases of AIDS-NHLs representing four tumor types: BL (n = 6), DLBCL (n = 8), PCNSL (n = 5), and PEL (n = 5). Also, in order to establish a differentiation specific miRNA profile, we compared the miRNA profile of three tonsillar B cell subsets: naïve, germinal center (GC), and memory. Our results demonstrate that miRNAs from the miR-17-92 paralog clusters are selectively upregulated by B cells during the GC stage of differentiation and are also significantly overexpressed in all of the AIDS-NHL types we examined. Additionally, we show that miR-17, miR-106a, and miR-106b inhibit p21 expression in AIDS-BL and DLBCL, thus supporting an oncogenic role for these miRNAs in AIDS-NHL pathogenesis.

## Materials and Methods

### Ethics statement

An application for the use of all anonymized patient samples, including tonsils, peripheral blood, and archived tissues utilized in this study was submitted to the UCLA Institutional Review Board (IRB), which concluded that these activities did not involve human subjects, and therefore did not require IRB review or certification, or exemption from IRB review. Tonsil tissue specimens (excess surgical pathology materials) were obtained from the Translational Pathology Core Laboratory (TPCL) of the University of California Los Angeles (UCLA) Medical Center. Written consent was not obtained from those subjects from whom the tonsils specimens were obtained. The TPCL has approval from the UCLA IRB confirming that patient consent is not needed when the human tissue samples are provided to investigators in a completely anonymized fashion. Peripheral blood mononuclear cells (PBMC) from healthy donors were provided by the Virology Core of the UCLA AIDS Institute, which obtained these discarded specimens from the UCLA Blood and Platelet center. These PBMC were obtained with written informed consent, and the Virology Core has IRB approval for obtaining and distributing these specimens. The AIDS & Cancer Specimen Resource (ACSR), which provided primary AIDS-NHL samples, obtained written informed consent from all participants and have approved IRB protocols for this study at each collecting sites.

### Cell lines, tissues, and clinical specimens

The AIDS-Burkitt's cell line (2F7, ATCC CRL-10237) and a DLBCL line (Toledo, ATCC CRL-2631) were cultured in RPMI 1640 (Cellgro, Manassas, VA) supplemented with 10% fetal calf serum (Atlanta Biologicals, Lawrenceville, GA), 1% L-glutamine (Cellgro, VA), and 1% penicillin/streptomycin (Cellgro, VA). 293T cells (ATCC CRL-11268) were cultured similarly, but in DMEM (Invitrogen, Carlsbad, CA). All three cell lines used in this study, 2F7, Toledo, and 293T were obtained from the ATCC (www.atcc.org). PBMC and tonsil tissue specimens were provided without any indirect or direct patient identifiers and were completely anonymized. Twenty-four primary B cell tumors from HIV infected individuals were obtained from the AIDS & Cancer Specimen Resource (ACSR) repository (Table S1). Snap frozen specimens were obtained for six Burkitt's lymphomas, eight DLBCL, and two PEL. 3×10 µM sections from FFPE blocks were obtained for one DLBCL, five CNS and three PEL tumors.

### Isolation of tonsillar B cell subsets

Tonsils, obtained from four different donors, were minced and the mononuclear cell population was isolated from the buffy layer following centrifugation in Ficoll-paque (GE Healthcare, Uppsala, Sweden). B cells were isolated from this population using CD19+ immunomagnetic dynabeads (Invitrogen, Carlsbad, CA). The CD19+ immunomagnetic beads were removed using CD19 DETACHaBEAD (Invitrogen, Carlsbad, CA). These CD19+ cells were then stained with CD38-PE (BD Pharmingen, San Jose, CA) and IgD-FITC (BD Pharmingen, San Jose, CA) antibodies. Following staining, the cells were separated into naïve (IgD+, CD38−), germinal center (IgD−, CD38+), and memory (IgD−, CD38−) populations using the method described by Pascual V *et al.* [33]. Fluorescence-activated cell sorting (FACS) was performed using FACSAriaII high-speed cell sorter (Becton Dickinson) housed at the UCLA Jonsson Comprehensive Cancer Center (JCCC) and Center for AIDS Research Flow Cytometry Core Facility.

### Total RNA isolation

Total RNA from tonsillar B cell subsets, and cell lines were extracted using mirVana miRNA isolation kit (Ambion, Austin, TX); from snap-frozen tumor specimen using mirVana PARIS kit (Ambion, Austin, TX); and from FFPE samples using RecoverAll total nucleic acid isolation kit (Ambion, Austin, TX). All extraction was done following the manufacturer's suggested protocol.

### MicroRNA expression profiling

Cellular and viral miRNAs were profiled at the UCLA Clinical Microarray Core facility using miRCURY™ LNA microRNA array v.11.0 (Exiqon, Vedbaek, Denmark). Briefly, up to 0.5 µg total RNA was Hy3 labeled and hybridized overnight to the microRNA array. The following day, the array slides were washed and scanned using GenePix personal 4100A scanner (Axon Instruments, Union City, CA). After data quality evaluation and filtration to remove miRNAs with signals below baseline (mean of negative control+3 standard deviation), the signal was normalized using a factor derived from the mean of the signal intensity of several house-keeping small RNAs (U6, snRNAs, snoRNAs and 5SRNAs). This normalized data was then log2-transformed and analyzed using the MultiExperiment Viewer Software v.4 (http://mev.tm4.org) [34]. miRNA expression was ranked by standard deviation across samples and only the top 50% of variably expressed miRNAs were selected for further analysis. Unsupervised hierarchical clustering was done using Euclidean distance and average linkage clustering. This microarray data is available through the Gene Expression Omnibus (GEO) database (http://www.ncbi.nlm.nih.gov/geo) using accession number GSE27504.

### MicroRNA target prediction

Predicted gene targets of miRNAs were obtained from the TargetScan [35] website: http://www.targetscan.org.

### Real-time quantitative PCR

Mature miRNAs miR-17, miR-18a, miR-19a, miR-106a, and miR-106b were first converted to cDNAs using TaqMan microRNA RT kit and microRNA specific primers (Applied Biosystems, Foster City, CA) followed by quantitative PCR using TaqMan microRNA Assay (Applied Biosystems, Foster City, CA). Sequences for all miRNAs that are targeted by these assays can be found at: http://www.appliedbiosystems.com/. miRNA levels were normalized to the expression of small nucleolar RNA, RNU48. Relative mRNA levels of p21 were quantified using the one step TaqMan RNA to C_T_ reagent (Applied Biosystems, Foster City, CA) in combination with gene specific primers/probe from TaqMan gene expression assay (Applied Biosystems, Foster City, CA). All mRNA expression values were normalized to β-actin levels. C_T_ values were obtained using ABI 7300 Real-Time machine (Applied Biosystems, Foster City, CA). The relative amount of miRNA (or mRNA) was calculated as 2^−dCT^ (where dCT = C_T gene(or miRNA)_−C_T endogenous control_) and expressed as a % of the endogenous control. The efficiency of PCR was calculated from the slope of the standard curve and was within the range of 90–110%.

### Cell transfection and proliferation assay

AIDS-BL line 2F7 and DLBCL line Toledo were transfected using Amaxa nucleofector reagent V (Lonza, Switzerland) using a nucleofector device (Lonza, Switzerland). Briefly, 5×10^6^ cells were resuspended in 100 uL of reagent V with synthetic miRNA precursors or anti-miRNAs (Ambion, Austin, TX) at a concentration of 200 nM. The cells were then electroporated using pre-set protocol O-006. Transfected cells were cultured in T25 flasks and cell proliferation was assessed by using XTT Cell Proliferation Kit II (Roche, Mannheim, Germany). Briefly, 100 µL aliquots of cell culture were incubated for 4 hours with a 50∶1 mixture of XTT labeling reagent∶electron-coupling reagent. Metabolization of XTT to formazan salt by viable cells was measured as absorbance value at 480 nm with 650 nm being the reference.

### Western blots

Cells were washed with 1× PBS and resuspended in RIPA buffer (20 mM Tris, pH 7.5, 150 mM NaCl, 1% Nonidet P-40, 0.5% sodium deoxycholate, 1 mM EDTA, 0.1% SDS) supplemented with 1× Halt protease inhibitor cocktail (Pierce, Rockford, IL) and 20 µM MG-132 (VWR, West Chester, PA). The cells were iced for 10 minutes, sonicated briefly, vortexed and centrifuged. The supernatant was collected and the protein quantified using BCA assay (Pierce, Rockford, IL). The cell lysates were run on 4–20% Tris-HCL ready gels (Biorad, Hercules, CA), transferred for 1.5 hrs on to Immobolin-P^sq^ membrane (Millipore, Billerica, MA) and blocked using 5% non-fat dry milk before incubating overnight in primary antibodies. Following washing, HRP conjugated secondary antibodies were added for 1 hour and the signal detected using Supersignal West Pico substrate (Pierce, Rockford, IL). The following antibodies were used at the concentration recommended by the manufacturers: p21 (DCS60), Rb (4H1), p53 (1C12), Bcl2 (#2872), CDK4 (DCS156), CDK6 (DCS83), and Bim (Cell Signaling Technology, Boston, MA); p53 (DO-7) (Dako, Carpinteria, CA) and β-actin (AC-15) from Sigma (St Louis, MO). Kodak 1D 3.6 Scientific Imaging Software was used to quantify protein band intensity. For each gene the normalized expression was calculated as: gene signal/β-actin signal where signal = mean pixel intensity×area of band.

### 3′UTR cloning and luciferase assays

The 3′UTR of CDKN1A was obtained by PCR of its cDNA clone (OriGene, Rockville, MD). *Spe* I and *Mlu* I restriction sites was introduced at its ends and then cloned into pMIR-REPORT vector (Ambion, Austin, TX) downstream of firefly luciferase. Additionally, to disrupt the miRNA:3′UTR interaction, two base pairs in the middle of the miRNA recognition seed sequence were mutated using the QuickChange II XL site-directed mutagenesis kit (Stratagene, La Jolla, CA). All inserts and mutations were verified by sequencing.

The following primers were used (restriction sites are *italicized*, mutations are in **bold**): CDKN1A 3′UTR Fw GCTG*ACTAGT*CACAGGAAGCCTGCAGTCCT, Rv CGAC*ACGCGT*GAGCACCTGCTGTATATTCAGC; CDKN1A Fw (CT to GA mutation, site #1, nucleotide 468–474).

TTTGAGAAGTAAACAGATGGCA**GA**TTGAAGGGGCCTCACCGAGTG and reverse compliment; CDKN1A Fw (CT to GA mutation in site#2, nucleotide1148–1154).

CATCCCTCCCCAGTTCATTGCA**GA**TTGATTAGCAGCGGAACAAGG and reverse compliment.

Briefly, 293T cells were seeded at a density of 50,000 cells/well in 24 well plates a day before transfection in antibiotic-free media. 20 ng of the plasmids with the 3′UTR of the gene of interest cloned into the pMIR-REPORT were co-transfected with 10 ng of renilla luciferase expressing vector pRL-SV40 (Promega, Madison, WI) and 100 nM precursor/antimiR miRNA oligos (Ambion, Foster City, CA) using lipofectamine-2000 reagent (Invitrogen, Carlsbad, CA). 24 hours post transfection, cells were washed with PBS and the amount of firefly and renilla luciferase was quantified using dual-luciferase reporter assay system (Promega, Madison, WI) using a BD monolight 2010 instrument (BD, New Jersey). All samples were assayed in triplicates and independently repeated three times.

### Statistical analysis

Comparison between groups was done by t-test or Mann-Whitney U test using the GraphPad Prism 5 software. p<0.05 was considered statistically significant. Error bars represent standard deviation.

## Results

### miRNAs are differentially expressed in tonsillar B cell subsets

**Figure 1:**
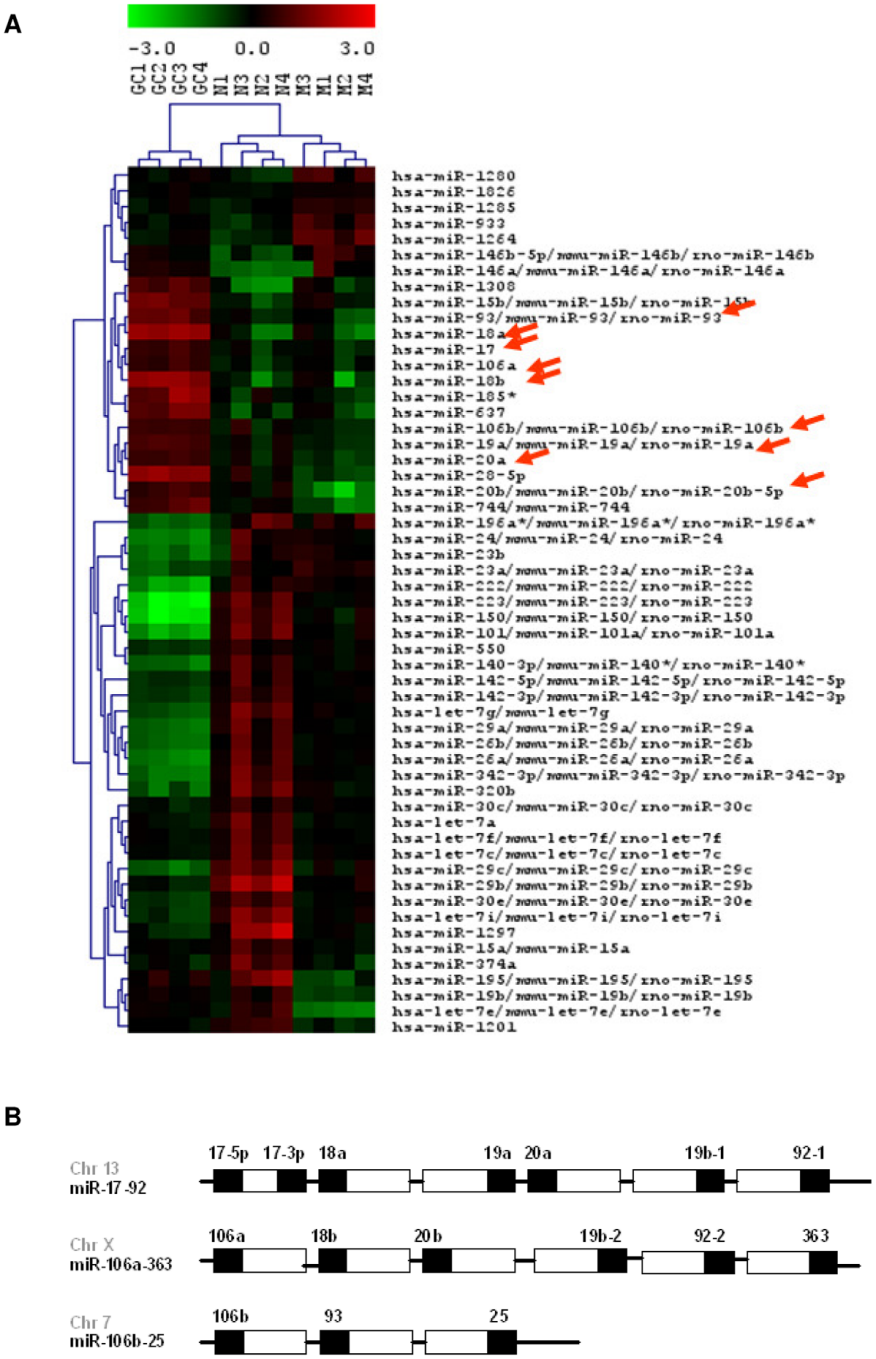
miRNA profiling of tonsillar B cell subsets. (**A**) Unsupervised hierarchical clustering of miRNAs from naïve (N) (IgD+, CD38−), germinal center (GC) (IgD−, CD38+), and memory (M) (IgD−, CD38−) cells isolated from CD19+ tonsillar B cells. Arrows point to miRNAs that are members of the miR-17-92 paralog clusters. Red denotes overexpression; green, downregulation; and black, median expression. (**B**) Schematic representation of the three paralogous cluster showing genomic location and the relative position of individual miRNAs (figure modified from Tanzer A *et al.* [61]).

Most cancers of mature B cells, including many AIDS-NHL, are thought to arise from neoplastic transformation during the GC transition. Hence, in order to establish a miRNA signature associated with normal B cell differentiation and maturation we first looked at the miRNA expression pattern of tonsillar B cell subsets. Mature B cells isolated from tonsils were separated by flow cytometry into three subsets: naïve (IgD+, CD38−), germinal center (GC) (IgD−, CD38+), and memory (IgD−, CD38−) B cells. miRNA expression in these B cell subsets was profiled using Exiqon's miRCURY™ LNA microRNA microarray. Unsupervised hierarchical clustering of miRNAs from these subsets produced a heat map, which demonstrates that these B cell subsets cluster distinctly from each other (Figure 1A). Out of the 48 miRNAs differentially regulated between the naïve to GC transition, 33 miRNAs were downregulated and 15 miRNAs were distinctly upregulated. Several miRNAs including miR-30, miR-150, miR-222, miR-223, and the let-7 family of miRNAs (let-7a, let-7c, let-7e, let-7f, let-7g, and let-7i) were downregulated by the naïve B cell subset upon GC entry, and remained at baseline levels even in the memory subsets. Among those 15 GC upregulated miRNAs, eight miRNAs (miR-17, miR-18a, miR-18b, miR-19a, miR-20a, miR-20b, miR106a, miR-106b) are members of the miRNA paralog clusters miR-17-92, miR106a-363, and miR-106b-25, located at three different chromosomal sites (Figure 1B). Six miRNAs (miR-1280, miR-1826, miR-1285, miR-933, miR-1264, miR-146b-5p) were also found to be specifically upregulated in memory subsets. Overall, tonsillar B cell subsets show a remarkable level of coregulation of miRNAs as groups in an asymmetrical fashion, especially that of the miR-17-92 paralog clusters (indicated by arrows in Figure 1A), which is GC subset specific.

### miRNAs from the miR-17-92 paralog clusters are overexpressed in AIDS-NHLs

**Figure 2:**
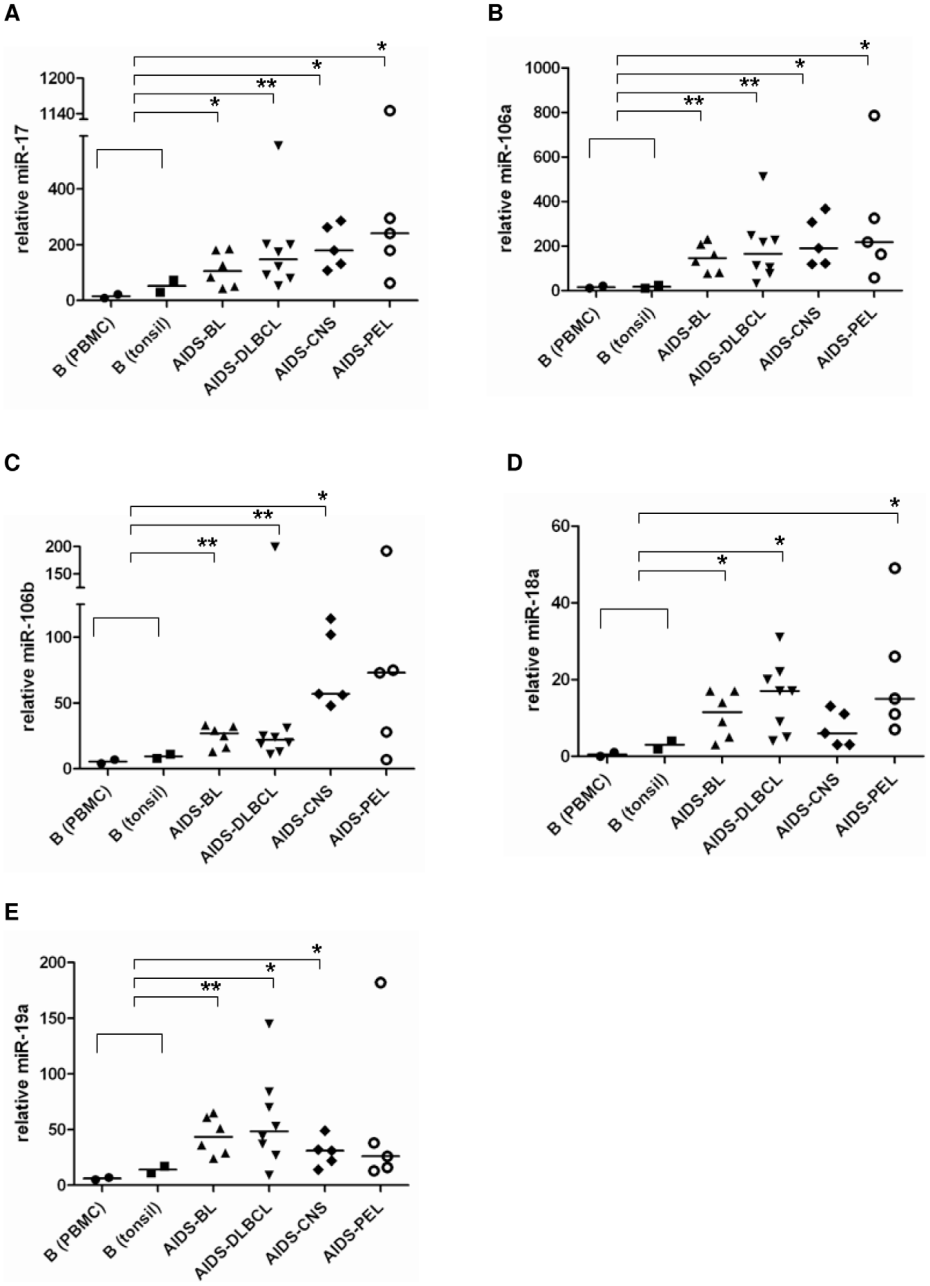
miRNAs from miR-17-92, miR-106a-363, and miR-106b-25 paralogous clusters are overexpressed in AIDS-NHLs. Taqman q-PCR assessed relative levels of (**A**) miR-17, (**B**) miR-106a, (**C**) miR-106b, (**D**) miR-18a, and (**E**) miR-19a in AIDS related BL, DLBCL, CNS, PEL compared to B cells from PBMC and tonsils. The expression of miRNAs was normalized to the expression of RNU48. Table S2 summarizes the Y-axis values used in these plots. Horizontal line through sample cluster represents the median value for each group. The median of each tumor group was compared to the combined median of the non-tumor group (PBMC and tonsil). P values were calculated using two-tailed Mann-Whitney U test (* signifies p<0.05 and ** signifies p<0.01). BL, Burkitt's lymphoma; DLBCL, diffuse large B-cell lymphoma; CNS, primary central nervous system lymphoma; PEL, primary effusion lymphoma.

Our study of AIDS-NHL includes mature B cell lymphoma types derived from GC or post-GC B cells. BL and DLBCL display GC B cell markers. PCNSL are DLBCL with immunoblastic features and EBV LMP1 expression. Both PCNSL and PEL display post-GC markers. Based on this information and our data showing specific upregulation of the miR-17-92 paralog clusters in GC subsets, we used quantitative realtime PCR (q-PCR) to assess the level of several miRNAs encoded by this cluster in 24 primary AIDS-NHL samples (Table S1). The expression of miRNAs was normalized to the expression of the small nucleolar RNA, RNU48, and shown as relative miRNA levels (Table S2). We assessed the expression of miR-17, miR-106a, miR-106b, miR-18a, and miR-19a in AIDS-NHL subtypes, BL (n = 5), DLBCL (n = 8), PCNSL (n = 5), PEL (n = 5) and compared it to control CD19+ B cells isolated from PBMC and tonsils (Figure 2 A–E). In the control samples, as expected, there was a clear (but nonsignificant) elevation of these miRNAs in tonsillar B cells compared to peripheral B cells. However, all of these miRNAs were significantly overexpressed in the lymphoma samples (*p<0.05; **p<0.01) when compared to non-neoplastic B cell controls (both, PBMC and tonsil derived), except for in three instances: miR-106b in PEL (p = 0.085), miR-18a in PCNS (p = 0.0651), and miR-19a in PEL (p = 0.0635) (Figure 2 A–E). Nonetheless, even in the nonsignificant cases, the mean (data not shown) and median of these miRNA expressions was clearly higher than in the controls. Overall, our results show that miRNAs from the miR-17-92 paralog clusters—miR-17, miR-106a, miR-106b, miR-18a, and miR-19a are overexpressed in AIDS-NHLs.

### miR-17 family members target p21/CDKN1A

**Figure 3:**
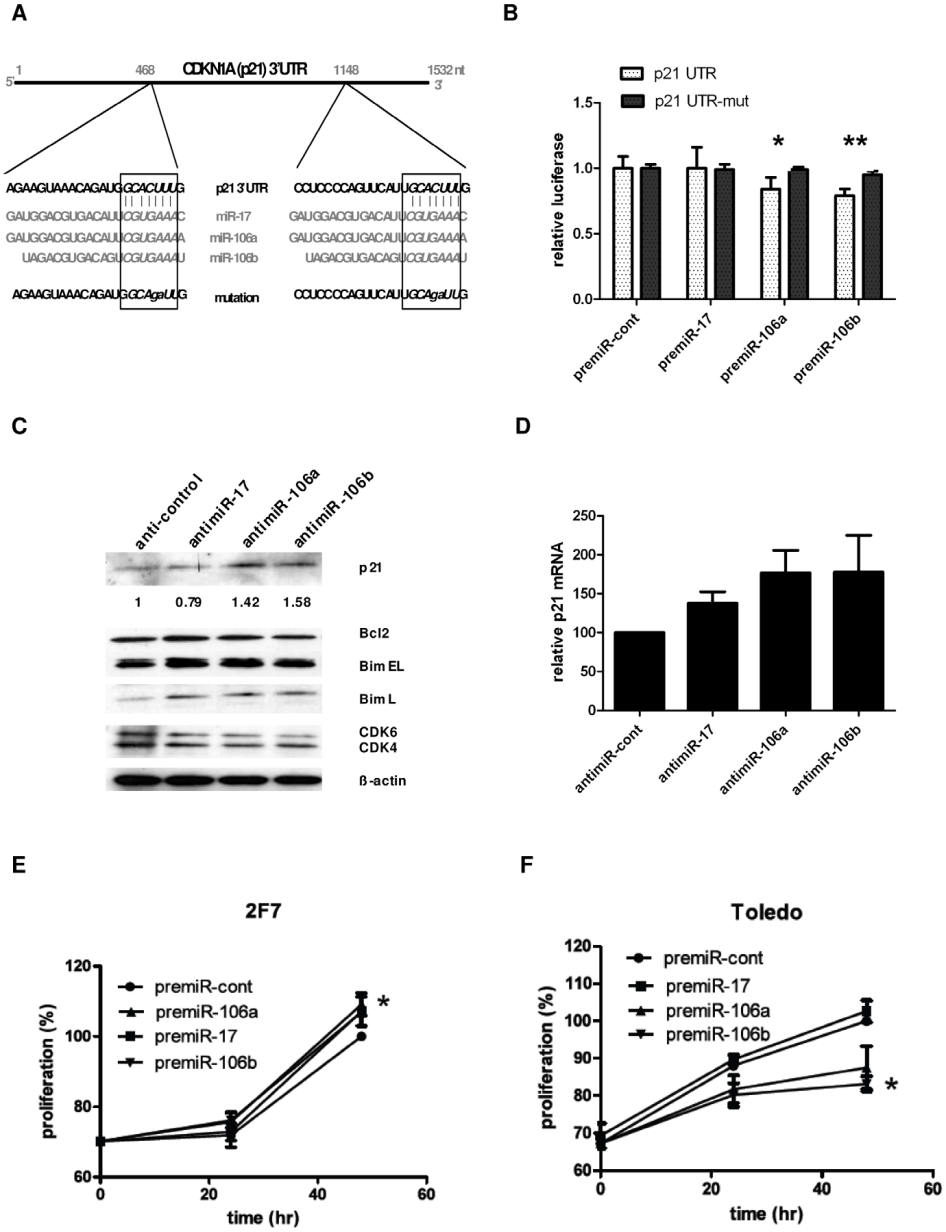
miR-17 family members target p21/CDKN1A. (**A**) Schematic of the 3′UTR of p21 showing the two locations in the 3′UTR targeted by miR-17 family. Boxed region shows the miRNA seed sequence common to this family. Mutations made in the p21 3′UTR construct that was cloned into p-MIR-REPORT vector is also shown. (**B**) p21-3′UTR firefly/renilla luciferase reporter activity in 293T cells cotransfected with precursor miRNAs. premiR-106a (*, p = 0.03) and premiR-106b (**, p = 0.01) showed significant downregulation of luciferase activity when wt p21-UTR was compared to p21-UTR mutant. Error bars represent the mean +/− SD of three independent experiments. Relative luciferase = Firefly luciferase (3′UTR)/Renilla luciferase (transfection control). (**C**) Representative Western blot of Toledo cells 3 day post transfection with antimiRs-17, -106a, -106b. Numbers represent fold change in p21 protein density (normalized to β-actin). antimiR-control transfected lane value was set to 1. The membrane was stripped and reprobed sequentially for Bcl2, Bim, CDK 4/6, and β-actin. (**D**) Taqman q-PCR of p21 mRNA levels normalized to β-actin in Toledo cells transfected as in C. Error bars represent mean +/− SD of three independent experiments. (**E, F**) 2F7 and Toledo cells were transfected with precursors or antimiRs (respectively) for miR-17, miR-106a, and miR-106b along with scrambled controls. XTT cell proliferation assay was done at 24 hr intervals post transfection. Proliferation of cells were standardized to the proliferation of control pre/antimiR transfected cells at 48 hrs, which was set to 100%. Error bars represent the mean +/− SD of three independent experiments. * signifies p<0.05 for miR-106b when compared to control proliferation.

Given the overexpression of miR-17-92 cluster miRNAs in AIDS-NHL, we next focused on potential molecular targets for these miRNAs. miRNAs target mRNAs with a specificity primarily determined by Watson-Crick base-pairing of the miRNA seed region (nucleotides 2 to 7/8) to the 3′UTR of target mRNAs. Hence, miRNAs sharing similar seed sequences are grouped as families and they are predicted to have similar targets. As such, we selected miR-17, miR-106a, and miR-106b which all share the seed sequence AAAGUG, for further analysis. TargetScan (http://www.targetscan.org) predicts 2 binding sites for this family of miRNAs in the 3′UTR of the key cell cycle inhibitor p21 (Figure 3A). We cloned the 3′UTR of p21 with, or without, mutation in both seed region recognition sites downstream of the firefly luciferase gene. This construct was then co-transfected into 293T cells with renilla luciferase (as transfection control) and precursor miRNAs. For bona fide miRNA targets, it is expected that miRNA:mRNA interaction will lead to suppression of firefly luciferase activity. miR-106a and miR-106b significantly inhibited luciferase activity in unmodified p21 3′UTR when compared to the mutant p21 3′UTR (Figure 3B). In order to shown that this miRNA:mRNA interaction also takes place in a physiological milieu in B cells, we transfected antimiRs to specifically block the endogenous miR-17, miR-106a, and miR-106b in Toledo cells. Functionally blocking a miRNA should lead to decreased binding of the miRNA to its target mRNA, and consequently, increased target protein expression. As expected, blocking endogenous miR-106a and miR-106b activity in Toledo resulted in a 1.4 and 1.6 fold increase in p21 protein levels respectively (Figure 3C). This increase was also reflected at the mRNA level with a 1.77 fold increase in p21 mRNA for both antimiR-106a and antimiR-106b (Figure 3D). However, blocking endogenous miR-17 did not increase p21 protein level (in fact, a 0.79 fold decrease was noted) (Figure 3C), despite a 1.37 fold increase in p21 mRNA level (Figure 3D). This suggests that, while miR-17 has some affinity for p21 mRNA binding, the overall p21 protein expression may be primarily dependent on the presence of miR-106a and miR-106b, which presumably bind p21 mRNA with a higher affinity.

Additionally, we also looked at the effect that blocking these miRNAs have on the expression of other proteins that play a role in cell survival and proliferation. Inhibition of all three of these miRNAs had no effect on the levels of Bcl2, but led to increased levels of Bim (isoform L), and decreased levels of CDK4 and CDK6 (Figure 3C). Both Bcl2 and Bim regulate apoptosis, albeit in an opposing manner. Bcl2 is a pro-survival molecule that inhibits apoptosis while Bim is a proapoptotic molecule. CDK4 and CDK6 promote S phase entry and cell cycle progression by partnering with cyclin D to phosphorylate the Rb protein. Overall, these results demonstrate an oncogenic role for miR-17, miR-106a, and miR-106b by targeting the cell cycle inhibitor p21 and the proapoptotic Bim protein.

Given that these miRNAs target a cell cycle inhibitor, next we studied its effect on cellular proliferation. We selected two cell lines, 2F7 (AIDS-BL) with modest expression and Toledo (DLBCL), with overexpression, of miR-17, miR-106a, and miR-106b. Overexpression of synthetic precursors for all three miRNAs increased cellular proliferation in 2F7 cells by 48 hrs post transfection (Figure 3E). We also transfected synthetic antimiRNAs to functionally block these endogenous miRNAs in Toledo cells. AntimiR-17 showed no effect in Toledo proliferation, whereas proliferation was decreased with antimiR-106a and antimiR-106b by up to 87% and 83% of control proliferation, respectively (Figure 3F). Thus, miR-17, miR-106a, and miR-106b accelerate cellular proliferation by targeting the cell cycle inhibitor p21.

### p21 is silenced post-transcriptionally in primary AIDS-NHL tumors

**Figure 4:**
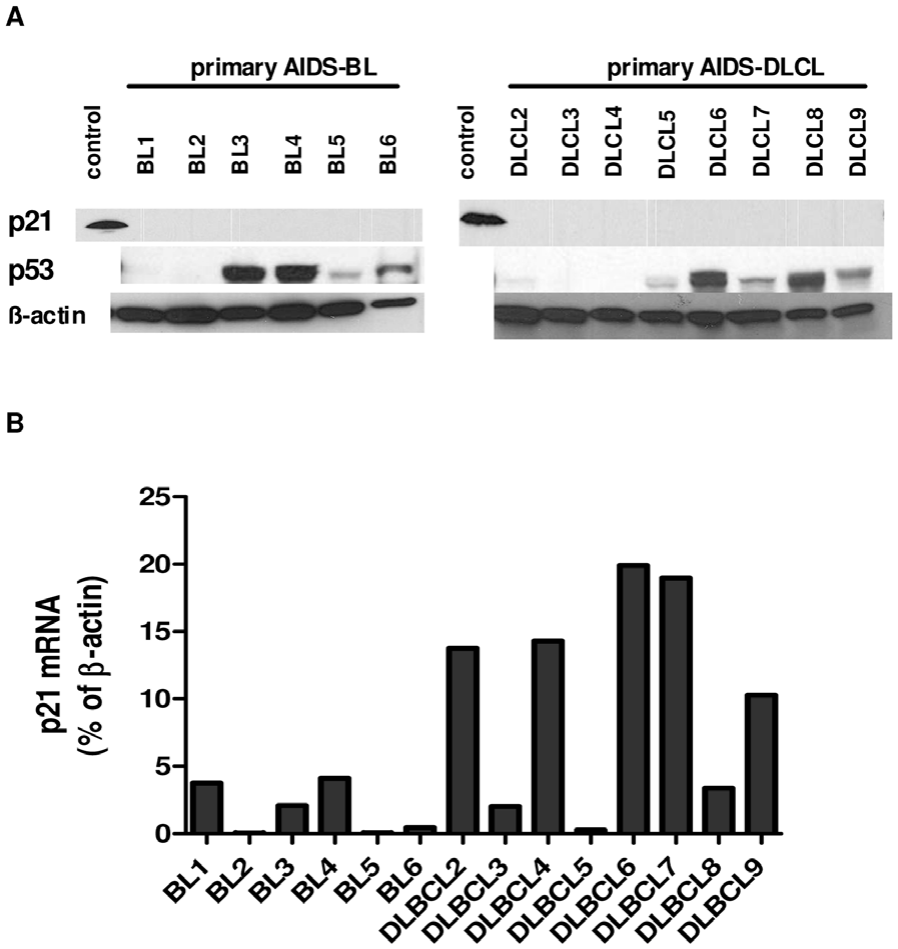
p21 protein and RNA expression in primary AIDS-NHL samples. (**A**) p21 Western blot analysis of primary AIDS- BL and AIDS-DLBCL samples. The blot was stripped and sequentially probed for p53 (Ab clone DO-7) and β-actin. No p21 protein expression was detected. The positive control is lysate from p21 cDNA transfected B cell line Ramos. (**B**) Taqman q-PCR of p21 mRNA levels in primary AIDS-NHL samples shown in A. In each sample, p21 mRNA level is shown as a % of β-actin expression, which is set to 100.

Finally, we examined p21 protein (Figure 4A) and mRNA (Figure 4B) expression in primary AIDS-BL and AIDS-DLBCL cases. p21 mRNA levels in Figure 4B are shown as a percentage of the housekeeping gene β-actin expression (set to 100). p21 protein expression was not detected in any of the tumor samples, despite p21 mRNA levels approaching up to 20% of β-actin expression. We also looked at the expression of p53 in these tumors using the anti-p53 antibody clone D0-7 which detects both wild-type and the mutant p53 variant (Figure 4A). p53, in the setting of DNA damage, induces p21 expression at the transcriptional level. p53 expression was detected in four of six BL and five of eight DLBCL tumors (Figure 4A). However, inactivating mutations in p53 occurs in up to 60% of AIDS-BL [36] and 40% of AIDS-DLBCL [37] often leading to enhanced p53 protein stability and its accumulation in tumors [38]. Therefore, it is likely that we are detecting mutant p53, which is unable to act as a transcription factor, thus having no effect on p21 transcription. Overall, due to the lack of p21 protein detection we are unable to show an inverse correlation between these miRNAs and p21 protein expression. However, given the p21 mRNA expression in these tumors, it is clear that miRNA-17 family members (including miR-17, miR-106a, miR-106b) play a significant role in inhibiting p21 protein expression in AIDS-NHLs.

## Discussion

Recently, it has become increasingly clear that miRNAs play a significant role in B cell tumorigenesis, especially those encoded by the miR-17-92 polycistron. However, the role of miRNAs in NHL in the setting of HIV/AIDS has only begun to be appreciated. Previous studies have found downregulation of the tumor suppressor miRNAs, miR-221, miR-222, and let-7 in PEL lines [39], [40], and targeting of the T-cell attracting chemokine CXCL-11 by EBV miRNA BHRF1-3 in primary AIDS tumors [41]. In this report, we used primary AIDS-NHL tumors and investigated whether miRNAs from the miR-17-92 cluster and its paralogs were also associated with AIDS-NHLs pathogenesis.

First, in order to establish a differentiation specific role for miRNA in normal B cell maturation, we assessed the miRNA profile of naïve, GC, and memory B cells isolated from tonsils. We and others [42]–[44] have shown that these B cell subsets exhibit a distinct miRNA signature. Many of the miRNAs that are dynamically regulated as the cells progress through the GC reaction have been shown to target transcription factors and key regulators of cell proliferation and apoptosis. miR-223, which is downregulated during the naïve to GC transition, targets the GC expressed transcription factors LMO2 [42], [43] and MYBL1 [43]. miR-150, downregulated in GC subsets, targets c-Myb and survivin [44]. miR-30c and miR-30e are downregulated in GC when its target, the GC specific Bcl6 is expressed [45]. And let-7a, which is expressed only in the naïve subset, targets PRDM1/Blimp-1 [46], which is a key regulator of post GC differentiation into plasma cells. Finally, we show that miRNA members from the miR-17-92 paralog clusters (miR-17, miR-18a, miR-18b, miR-19a, miR-19b, miR-20a, miR-20b, miR-106a, and miR-106b) are tightly controlled in normal B cells, with increased expression seen only during naïve to GC transition and immediate downregulation upon GC exit (Figure 1A).

The oncogenic role for the miR-17-92 polycistron in B cell NHL has been well established [19]–[23], [30]. Gene duplication events of the miR-17-92 cluster (chromosome 13) gave rise to its paralogs, miR-106a-363 (chromosome X), and miR-106b-25 (chromosome 7) (Figure 1B). We find that miRNAs from all three clusters, miR-17, miR-18a, miR-19a from miR-17-92 cluster; miR-106a from miR-106a-363 cluster; and miR-106b from miR106b-25 cluster, are overexpressed in various AIDS-NHLs including, BL, DLBCL, PCNSL, and PEL types. miRNA members from this paralog clusters have been shown to target genes involved in cell cycle/proliferation and apoptosis. miR-17-5p and miR-20a target cyclin D1 [47]; miR-25 targets Bim [48] and p57 [49]; miR-17 and miR-20 target E2F1 [50]; miR-19a targets PTEN [51]; and miRNAs-17, miR-20, miR-106b, and miR-93 target p21 [27], [28], [48], [52]. Recently, p21 was also shown to be targeted by the PEL associated herpesvirus HHV-8 miR-K1 [53]. p21 is a cyclin dependent kinase inhibitor that blocks G1 to S cell cycle progression. Our results show that the miR-17 family mediated suppression of p21 is also a common feature in AIDS-NHL. It is appropriate that miRNAs specifically upregulated during the GC stage target negative regulators of cell cycle progression. Rapid proliferation is a hallmark of GC B cells and is required for the generation of highly antigen specific plasma and memory cells. We hypothesize that the inability of normal B cells to turn off expression of miR-17-92 and its paralogs upon GC exit may be a factor in the pathogenesis of these mature B cell cancers.

Several studies have looked at the transcriptional regulation of the miR-17-92 cluster. *c-Myc*, the proto-oncogene activated in most BL, has been shown to be a transcription factor for the miR-17-92 polycistron [54]. c-Myc was also shown to have a significantly higher transcriptional activity in DLBCL tumor subsets overexpressing the miR-17-92 cluster miRNAs [55]. However, c-Myc is downregulated in centroblastic B cells in the germinal center [56] arguing for a distinct mechanism of miR-17-92 transcriptional control in normal GC B cells compared to tumors. E2F1 and E2F3, belonging to the E2F family of transcription factors required for cell cycle progression, have also been shown to activate miR-17-92 transcription [57]. And under hypoxic conditions, p53 directly competes with the TATA-binding protein (TBP) transcription factor binding to the promoter of miR-17-92, thus inhibiting its expression [58]. The precise mechanism by which these and other factors control transcription of miR-17-92 and its paralogs, leading to its dysregulation in various lymphoma subtypes, remain to be answered.

Finally, in addition to elucidating molecular roles for miRNAs in tumor pathogenesis, the possible utility of miRNAs as diagnostic and prognostic biomarkers cannot be overlooked, especially as these miRNAs can be detected in body fluids, which can be obtained by less invasive means than are required to obtain tumor biopsy specimens. Lawrie *et al.* reported the expression of miR-155, miR-210, and miR-21 in serum from DLBCL patients and found association of high miR-21 expression with relapse-free survival [59]. More recently, miR-21, and two members of the miR-17-92 cluster, miR-19 and miR-92a, were found to be elevated in the cerebrospinal fluid (CSF) of PCNSL patients [60]. This is in agreement with our results, which show overexpression of miR-17-92 paralog clusters in PCNSL tumors.

In conclusion, we show that miRNAs from the miR-17-92 cluster and its paralogs are overexpressed in various AIDS-NHL subtypes and provide evidence that these miRNAs contribute to the pathogenesis of these tumors by suppression of p21. Future studies looking at tumor deregulated miRNAs and its presence in relatively accessible body fluids should enhance the early detection, differential diagnosis, and prognosis of various B lymphoma subtypes.

## Supporting Information

Table S1 AIDS-NHL patient and tumor characteristics.
(XLS)

Table S2 q-PCR primary data for miRNA expression levels in AIDS-NHL cases and controls.
(XLS)

## References

[1] Raphael M, Borisch B, Jaffe ES, Jaffe ES, Harris NL, Stein H, Vardiman JW (2001). Lymphomas associated with infection by the human immune deficiency virus (HIV).. World Health Organization Classification of Tumors, Pathology and Genetics of Tumours of Haemotopoietic and Lymphoid Tissues.

[2] Crum-Cianflone N, Hullsiek KH, Marconi V, Weintrob A, Ganesan A (2009). Trends in the incidence of cancers among HIV-infected persons and the impact of antiretroviral therapy: a 20 year cohort study.. AIDS.

[3] Kirk O, Pedersen C, Cozzi-Lepri A, Antunes F, Miller V (2001). Non-Hodgkin lymphomas in HIV-infected patients in the era of highly active antiretroviral therapy.. Blood.

[4] Gaidano G, Carbone A, Dalla-Favera R (1998). Pathogenesis of AIDS-related lymphomas: molecular and histogenetic heterogeneity.. Am J Pathol.

[5] Bartel DP (2004). MicroRNAs:genomics, biogenesis, mechanism, and function.. Cell.

[6] Griffiths-Jones S, Grocock RJ, Van Dongen S, Bateman A, Enright AJ (2006). miRBase: microRNA sequences, targets and gene nomenclature.. Nuc Acids Res.

[7] Calin GA, Croce CM (2006). MicroRNA signatures in human cancers.. Nature Rev.

[8] Cimmino A, Calin GA, Fabbri M, Iorio MV, Ferracin M (2005). miR-15 and miR-16 induce apoptosis by targeting BCL2.. Proc Natl Acad Sci USA.

[9] Chen RW, Bemis LT, Amato CM, Myint H, Tran H (2008). Truncation in CCND1 mRNA alters miR-16-1 regulation in mantle cell lymphoma.. Blood.

[10] Tam W, Ben-Yehuda D, Hayward WS (1997). bic, a novel gene activated by proviral insertions in avain leukosis virus-induced lymphomas, is likely to function through its noncoding RNA.. Mol Cell Biol.

[11] Kluiver J, Poppema S, de Jong D, Blokzijl T, Harms G (2005). BIC and miR-155 are highly expressed in Hodgkin, primary mediastinal and diffuse large B cell lymphomas.. J Pathol.

[12] van den Berg A, Bart-Jan K, Dooistra K, de Jong D, Briggs J (2003). High expression of B-cell receptor inducible gene BIC in all subtypes of Hodgkin Lymphoma.. Genes Chr Cancer.

[13] Eis PS, Tam W, Sun L, Chadburn A, Li Z (2005). Accumulation of miR-155 and BIC RNA in human B cell lymphomas.. Proc Natl Acad Sci USA.

[14] Kluiver J, Haralambieva E, de Jong D, Blokzijl T, Jacobs C (2006). Lack of BIC and microRNA miR-155 expression in primary cases of Burkitt lymphoma.. Genes Chr Cancer.

[15] Costinean S, Zanesi N, Pekarsky Y, Tili E, Volinia S (2006). Pre-B cell proliferation and lymphoblastic leukemia/high-grade lymphoma in E(mu)-miR155 transgenic mice.. Proc Natl Acad Sci USA.

[16] Jiang J, Lee EJ, Schmittgen TD (2006). Increased expression of microRNA-155 in Epstein-Barr virus transformed lymphoblastoid cell lines.. Genes Chr Cancer.

[17] Skalsky RL, Samols MA, Plaisance KB, Boss IW, Riva A (2007). Kaposi's sarcoma-associated herpesvirus encodes an ortholog of miR-155.. J Virol.

[18] Gottwein E, Mukherjee N, Sachse C, Frenzel C, Majoros WH (2007). A viral microRNA functions as an orthologue of cellular miR-155.. Nature.

[19] Ota A, Tagawa H, Karnan S, Tsuzuki S, Karpas A (2004). Identification and characterization of a novel gene, C13orf25, as a target for 13q31-q32 amplification in malignant lymphoma.. Cancer Res.

[20] Rao PH, Houldsworth J, Dyomina K, Parsa NZ, Cigudosa JC (1998). Chromosomal and gene amplification in diffuse large-B cell lymphoma.. Blood.

[21] He L, Thomson JM, Hemann MT, Hernando-Monge E, Mu D (2005). A microRNA polycistron as a potential human oncogene.. Nature.

[22] Neat MJ, Foot N, Jenner M, Goff L, Ashcroft K (2001). Localisation of novel region of recurrent amplification in follicular lymphoma to an approximately 6.8 Mb region of 13q32-33.. Genes Chr Cancer.

[23] Monni O, Oinonen R, Elonen E, Franssila K, Teerenhovi L (1998). Gain of 3q and deletion of 11q22 are frequent aberrations in mantle cell lymphoma.. Genes Chr Cancer.

[24] Mao X, Lillington D, Child F, Russell-Jones R, Young B (2002). Comparative genomic hybridization analysis of primary cutaneous B-cell lymphomas: identification of common genomic alterations in disease pathogenesis.. Genes Chr Cancer.

[25] Mi S, Zejuan L, Chen P, He C, Cao D (2009). Aberrant overexpression and function of the miR-17-92 cluster in *MLL*-rarranged acute leukemia.. Proc Natl Acad Sci U S A.

[26] Venturini L, Battmer K, Castoldi M, Schultheis B, Hochhaus A (2007). Expression of the miR-17-92 polycistron in chronic myeloid leukemia (CML) CD34^+^ cells.. Blood.

[27] Inomata M, Tagawa H, Guo Y-M, Kameoka Y, Takahashi N (2009). MicroRNA-17-92 down-regulates expression of distinct targets in different B-cell lymphoma subtypes.. Blood.

[28] Wong P, Iwasaki M, Somervaille TCP, Ficara F, Carico C (2010). The miR-17-92 microRNA polycistron regulates MLL leukemia stem cell potential by modulating p21 expresssion.. Cancer Res.

[29] Mu P, Han Y-C, Betel D, Yao E, Squatrito M (2009). Genetic dissection of the miR-17-92 cluster of microRNAs in Myc-induced B-cell lymphomas.. Genes & Development.

[30] Xiao C, Srinivasan L, Calado DP, Patterson HC, Zhang B (2008). Lymphoproliferative disease and autoimmunity in mice with increased miR-17-92 expression in lymphocytes.. Nature Immunol.

[31] Ventura A, Young AG, Winslow MM, Lintault L, Meissner A (2008). Targeted deletion reveals essential and overlapping functions of the miR-17 through 92 family of miRNA clusters.. Cell.

[32] Goedert JJ, Cote TR, Virgo P, Scoppa SM, Kingman DW (1998). Spectrum of AIDS-associated malignant disorders.. Lancet.

[33] Pascual V, Liu Y-J, Magalski A, Bouteiller Od, Banchereau J (1994). Analysis of Somatic Mutation in Five B Cell Subsets of Human Tonsil.. J Exp Med.

[34] Saeed AI, Sharov V, White J, Li J, Liang W (2003). TM4: a free, open-source system for microarray data management and analysis.. Biotechniques.

[35] Lewis BP, Burge CB, Bartel DP (2005). Conserved seed pairing, often flanked by adenosines, indicates that thousands of human genes are microRNA targets.. Cell.

[36] Ballerini P, Gaidano G, Gong JZ, Tassi V, Saglio G (1993). Multiple genetic lesions in acquired immunodeficiency syndrome-related non-Hodgkin's lymphoma.. Blood.

[37] Martin A, Flaman JM, Frebourg T, Davi F, El Mansouri S (1998). Functional analysis of the p53 protein in AIDS-related non-Hodgkin's lymphomas and polymorphic lymphoproliferations.. Br J Haematol.

[38] Villuendas R, Piris MA, Algara P, Sanchez-Beato M, Sanchez-Verde L (1993). The expression of p53 protein in non-Hodgkin's lymphomas is not always dependent on p53 gene mutations.. Blood.

[39] O'Hara AJ, Wolfgang V, Dittmer DP (2008). Gene alteration and precursor and mature microRNA transcription changes contribute to the miRNA signature of primary effusion lymphoma.. Blood.

[40] O'Hara AJ, Wang L, Dezube BJ, Harrington WJ, Damania B (2009). Tumor suppressor microRNAs are underrepresented in primary effusion lymphoma and Kaposi sarcoma.. Blood.

[41] Xia T, O'Hara A, Araujo I, Barreto J, Carvalho E (2008). EBV microRNAs in primary lymphomas and targeting of CXCL-11 by ebv-mir-BHRF1-3.. Cancer Res.

[42] Malumbres R, Sarosiek KA, Cubedo E, Ruiz JW, Jiang X (2009). Differentiation-stage-specific expression of microRNAs in B-lymphocytes and diffuse B-cell lymphomas.. Blood.

[43] Zhang J, Jima DD, Jacobs C, Fischer R, Gottwein E (2009). Patterns of microRNA expression characterize stages of human B-cell differentiation.. Blood.

[44] Tan LP, Wang M, Robertus JL, Schakel RN, Gibcus JH (2009). miRNA profiling of B-cell subsets: specific miRNA profile for germinal center B cells with variation between centroblasts and centrocytes.. Lab Invest.

[45] Lin J, Lwin T, Zhao JJ, Tam W, Choi YS (2010). Follicular dendritic cell-induced microRNA-mediated upregulation of PRDM1 and downregulation of BCL-6 in non-Hodgkin's B-cell lymphomas.. Leukemia Epub ahead of print.

[46] Nie K, Gomez M, Landgraf P, Garcia J-F, Liu Y (2008). MicroRNA-mediated down-regulation of PRDM1/Blimp-1 in Hodgkin/Reed-Sternberg cells: a potential pathogenetic lesion in Hodgkin lymphomas.. Am J Pathol.

[47] Yu Z, Wang C, Wang M, Li Z, Casimiro MC (2008). A cyclin D1/microRNA 17/20 regulatory feedback loop in control of breast cancer cell proliferation.. J Cell Biol.

[48] Kan T, Sato F, Ito T, Matsumura N, David S (2009). The miR-106b-25 polycistron, activated by genomic amplification, functions as an oncogene by suppressing p21 and Bim.. Gastroenterology.

[49] Kim Y-K, Yu J, T.S. H, Park S-Y, Namkoong B (2009). Functional links between clustered microRNAs: suppression of cell-cyle inhibitors by microRNA clusters in gastric cancer.. Nuc Acids Res.

[50] Pickering MT, Stadler BM, Kowalik TF (2009). miR-17 and miR-20a temper an E2F1-induced G1 checkpoint to regulate cell cycle progression.. Oncogene.

[51] Olive V, Bennett MJ, Walker JC, Ma C, Jiang I (2009). miR-19 is a key oncogenic component of mir-17-92.. Genes & Development.

[52] Ivanovska I, Ball AS, Diaz RL, Magnus JF, Kibukawa M (2008). MicroRNAs in the miR-106b family regulate p21/CDKN1A and promote cell cycle progression.. Mol Cell Biol.

[53] Gottwein E, Cullen BR (2010). A human herpesvirus microRNA inhibits p21 expression and attenuates p21-mediated cell cycle arrest.. J Virol.

[54] O'Donnell KA, Wentzel EA, Zeller KI, Dang CV, Mendell JT (2005). c-Myc regulated microRNAs modulate E2F1 expression.. Nature.

[55] Li C, Sang-Woo K, Rai D, Bolla AR, Adhvaryu S (2009). Copy number abnormalities, MYC activity, and the genetic fingerprint of normal B cells mechanistically define the microRNA profile of diffuse large B-cell lymphoma.. Blood.

[56] Klein U, Tu Y, Stolovitzky GA, Keller JL, Haddad J (2003). Transcriptional analysis of the B cell germinal center reaction.. Proc Natl Acad Sci U S A.

[57] Woods K, Thomson JM, Hammond SM (2007). Direct regulation of an oncogenic micro-RNA cluster by E2F transcription factors.. J Biol Chem.

[58] Yan H-L, Xue G, Mei Q, Wang Y-Z, Ding F-X (2009). Repression of the miR-17-92 cluster by p53 has an important function in hypoxia-induced apoptosis.. EMBO.

[59] Lawrie CH, Gal S, Dunlop HM, Pushkaran B, Liggins AP (2008). Detection of elevated levels of microRNAs in serum of patients with diffuse large B-cell lymphoma. .. Br J Haematol.

[60] Baraniskin A, Kuhnhenn J, Schlegel U, Chan A, Deckert M (2011). Identification of microRNAs in the cerebrospinal fluid as marker for primary diffuse large B-cell lymphoma of the central nervous system.. Blood: Epub ahead of print.

[61] Tanzer A, Stadler PF (2004). Molecular evolution of microRNA cluster.. J Mol Biol.

